# Predictors of nutritional recovery time and survival status among children with severe acute malnutrition who have been managed in therapeutic feeding centers, Southern Ethiopia: retrospective cohort study

**DOI:** 10.1186/s12889-015-2593-5

**Published:** 2015-12-21

**Authors:** Delelegn Yilma Gebremichael

**Affiliations:** Department of Public Health, Ambo University, College of Medicine and Health Sciences, P.O. Box 19, Ambo, Ethiopia

**Keywords:** Severe acute malnutrition, Therapeutic feeding centers, Inpatient

## Abstract

**Background:**

Malnutrition remains to be one of the most common causes of morbidity and mortality among children in developing countries. The prevalence of wasting in Ethiopia remained about 10 % for the past ten years. Mortality rate of children with severe acute malnutrition treated in inpatient set ups has remained unacceptably high.

**Methods:**

A retrospective cohort study was conducted in Southern Ethiopia. The study population were children with severe acute malnutrition aged from 6 to 59 months who have been managed at Karat and Fasha stabilization centers between September 30, 2013, and Sep. 29, 2014. The total sample size was 420 and pretested questionnaire was used. Kaplan Meier analysis was used to estimate time to nutritional recovery and Cox proportional-hazard regression analysis was carried out to determine independent predictors.

**Results:**

Nutritional recovery rate was 3.61 per 100 person day observations. Median nutritional recovery time was 22 and 29 days for edematous malnourished and severely wasted children respectively. The independent predictors of nutritional recovery rate were: stabilization center (AHR = 1.4, 95 % CI: 1.1–1.7), malnutrition status (AHR = 1.8, 95 % CI: 1.3–2.4), weight (AHR = 1.5, 95 % CI: 1.2–1.9), mid- upper arm circumference (AHR = 1.4, 95 % CI: 1.1–1.9), inpatient complications (AHR = 2.2, 95 % CI: 1.4–3.5) and did not lose edema within four days of inpatient treatment (AHR = 2.3, 95 % CI: 1.1–4.8).

**Conclusions:**

The findings of this study confirm the probability of surviving gets slimmer with inpatient complications and staying longer in stabilization centers. So, to prevent complications and enhance recovery rate due emphasis should be given in improving early detection and treatment of severely malnourished children in Ethiopia.

## Background

Malnutrition remains one of the most common causes of morbidity and mortality among children throughout the world [1]. Malnutrition responsible for 53 % of deaths among under-five children [2]. Severe acute malnutrition (SAM) affects 20 million under five children and contributes to more than 1 million child deaths globally each year [3]. The prevalence of wasting remained 10 % in the past ten years in Ethiopia [4–6]. Prevalence of wasting was 12 % in Southern Ethiopia [7] while it was 9 % in Jimma Town [8]. In the study area 8 % of children with SAM died [9], but TFCs to be effective mortality rate has to be less than 5 % [10]. Survival of children with SAM treated in outpatient set-ups have been improved [4, 11]. However, mortality rate of children with SAM treated in inpatient set ups has remained unacceptably high [12]. Such high mortality in inpatient units has been attributed to either co-morbidities [13], or to poor adherence to WHO therapeutic guidelines [14]. Still there is high prevalence of malnutrition in Ethiopia, despite the availability of treatment for children with SAM in TFCs. So, this study intended to estimate nutritional recovery time, determine the contextual factors of nutritional recovery rate and assess effectiveness of TFCs compared to minimum international standard set for management of SAM.

## Methods

The study used a retrospective cohort study design and the study population were children with SAM aged from 6 to 59 months who have been managed at Karat and Fasha TFCs in southern Ethiopia between September 30, 2013, and Sep. 29, 2014. Severely wasted was defined as weight for height less than −3 SD (or z scores), or less than 70 % of the median WHO growth standard, or mid upper arm circumference less than 110 mm and Edematous malnutrition was defined as symmetrical oedema involving at least the feet. Recovered: weight-for-height of more than or equal to 85 % of the median WHO growth reference, absence of bilateral pitting oedema and no medical complication [10].

Children in affected areas were screened for signs of SAM and diagnosed based on anthropometric measurement and examination of the feet for bilateral pitting edema. Children who fulfill the criteria for admission were admitted to the TFC. If the W/H was < 70 % of the median WHO growth standard, or if the MUAC was found to be less than 11 cm, or children with bilateral pedal edema were admitted. On admission, malnourished patients were assessed for hydration status, anemia and signs of infections. They were given oral dose of Vitamin A, Mebendazole, folic acid and a course of Amoxicillin for five days. Rehydration Solution for Malnutrition (ReSoMal) was used for treating dehydrated cases and drugs like Gentamicin, Chloramphenicol or Quinine were used based on causes of infections. Treatment of severe malnutrition was divided into three phases; the first phase (phase I), transition phase and phase II. In phase I, health workers resuscitated patients, treated for infections, restored electrolyte balance, and prevented hypoglycemia and hypothermia on indication. F75 milk (formula 75 that contains 75 kcal in 100 ml, minerals and proteins) was used during phase I treatment; malnourished cases who responded on treatment by return of appetite, beginning of loss of edema and no intravenous line or nasogastric tubes were transferred to transition phase to receive F100 (formula 100 that contains 100 kcal in 100 ml). Afterwards, cases were transferred to phase II after they gained good appetite (finish 90 % of F100 prescribed for transition phase) clear edema. In phase II treatment, F100 was used as much as the children could take and additional diet is recommended until they achieved weight for height, 85 % and above [10].

The sample size was calculated based on the assumption that type I error 5 %, power of 90 %, median survival time among exposed (severely wasted) 39 days and median survival time among non-exposed (edematous malnutrition) 27 days [9]. The sample size was calculated by the following formula which depends on the separate median survival times for the exposed and non-exposed [15]. The required patients in each group were calculated as follow.

$$ \begin{array}{l}\kern10em n={\left({Z}_{\alpha }+{Z}_{\beta}\right)}^2\left[\varPhi \left({\mu}_{\mathrm{E}}\right)+\varPhi \left({\mu}_{\mathrm{C}}\right)\right]/{\left({\mu}_{\mathrm{E}}^{-1}-{\mu}_{\mathrm{C}}^{-1}\right)}^2\hfill \\ {}\mathrm{where}\hfill \\ {}\kern6.5em \varPhi \left({\mu}_i\right)=\frac{T}{\mu_i^3}/\left[\frac{T}{\mu_i}-1+ \exp \left(-T/{\mu}_i\right)\right],i=\mathrm{C},\mathrm{E}\hfill \end{array} $$

C = median survival time for unexposed group (27 days), E = median survival time for exposed group (39 days), T = total time study subjects were recruited to the study (365 days), α = level of significance (0.05), Zα/2 = 1.96 at 95 % confidence interval, Power = 1-β = 90 %, Zβ = 1.28. *n* = minimum sample size required for each group was 189 and after adding 10 % for incomplete records the sample size was 210 for each group.

**Fig. 1:**
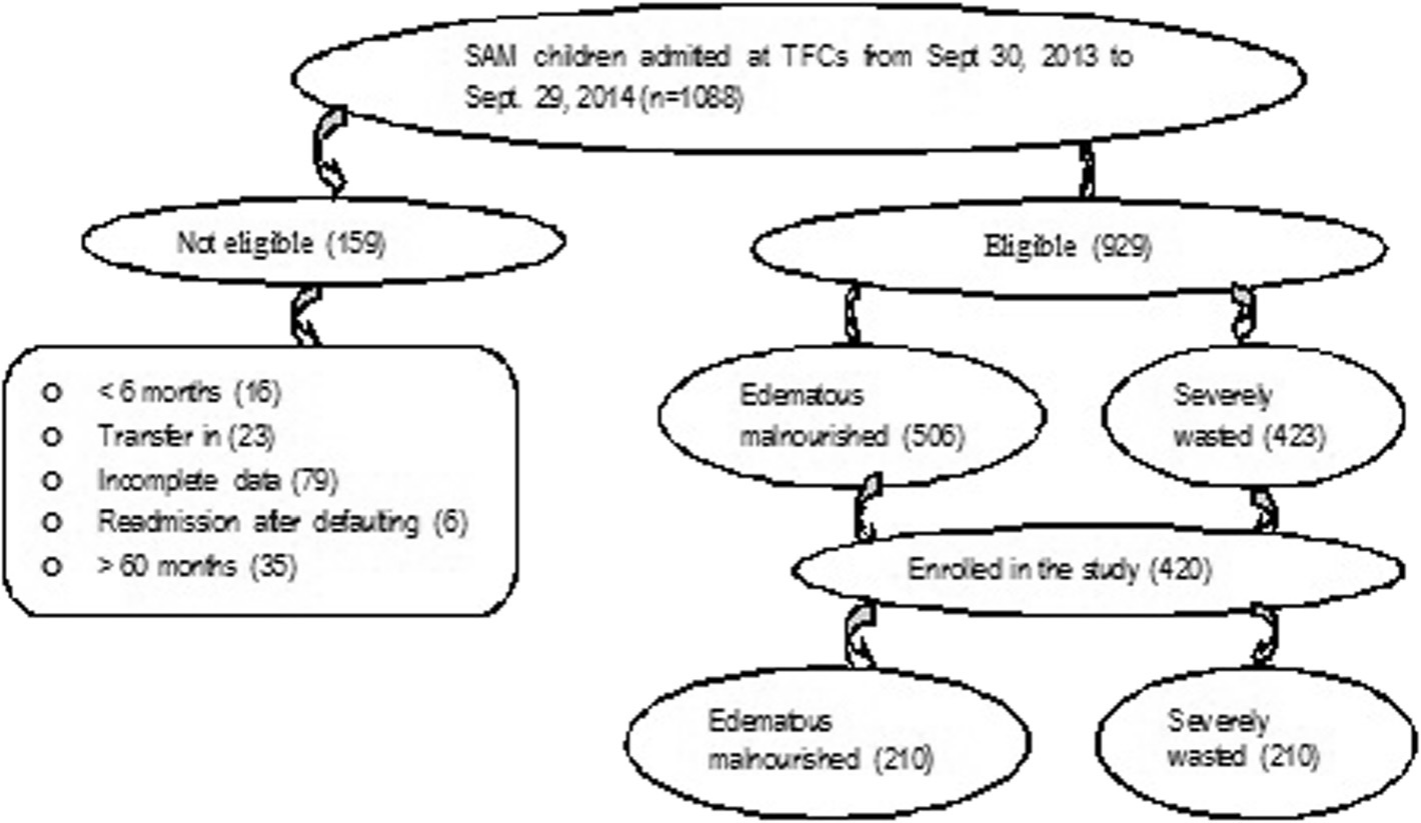
Schematic representation of sampling procedure in Karat and Fasha TFCs, Ethopia, 2013/14. Showed the process how study subjects were selected (sampling procedure). A total of 1088 children with SAM were admitted in Karat (664) and Fasha (424) TFCs; after evaluation, 159 of them were not fulfilled the inclusion criteria’s to be eligible to the study: 16 were under 6 months, 23 were Transferred from other TFCs, 79 had Incomplete data, 6 were readmitted after default and 35 were older than 60 months. 929 were eligible for the study. From 506 eligible edematous malnourished children using systematic sampling method with K = 2 interval 210 edematous children were selected. Similarly, from 423 eligible severely wasted children using systematic sampling method with K = 2 interval 210 severely wasted children were also selected

A total of 1088 children with SAM were admitted in Karat (664) and Fasha (424) TFCs; after evaluation, 929 were eligible for the study. From 506 eligible edematous malnourished children using systematic sampling method with K = 2 interval 210 edematous children were selected. Similarly, from 423 eligible severely wasted children using systematic sampling method with K = 2 interval 210 severely wasted children were also selected (Fig. 1).

The data were collected by reviewing records from inpatient therapeutic feeding registration book and individual follow-up chart using pretested data collection form. Data were entered to Epi-Info 3.5.1 for windows and analyzed using SPSS version 16 for windows and Stata version 11. The patient cohort characteristics were described in terms of mean (Standard deviation) and median (inter quartile range) for continuous data and frequency distribution for categorical data. Independent sample t-test and Chi square (*X*^2^) test were used for continuous and categorical variables respectively. Actuarial Life Table analysis was used to estimate cumulative proportion of survival among children with SAM at different time points. Kaplan Meier Survival Curve was used to estimate nutritional recovery time after initiation of inpatient treatment, and log rank test was used to compare time to nutritional recovery between groups. The relationship between nutritional recovery rate and covariates was analyzed using COX bivariate proportional regression model after proportional hazard assumption checked by examining Log (−Log) S (t) plots. In order to identify independent predictors of nutritional recovery rate a multivariate COX-proportional adjusted model was carried out. Multicolinearity was checked using variance inflation factor. Crude and adjusted hazard ratios with their 95 % Confidence Interval (CI) were estimated and *P*-Value less than 0.05 was used to declare presence of significant association between nutritional recovery rate and covariates.

Ethical approval was obtained from the Institutional Review Board (IRB) of Addis Ababa University, College of Health Sciences. Following the approval by IRB, official letter of co-operation was written to the concerned bodies by the School of Public Health, about the purpose of the study to facilitate the support and commitment of responsible bodies. As the study was conducted through review of medical records, the individual patients were not subjected to any harm and. personal identifiers were not used on data collection forms and confidentiality was maintained.

## Results

### Socio-demographic characteristics

Out of the total cohorts; 256 (61 %) were from Karat and 164 (39 %) were from Fasha TFCs. Majority of study subjects were rural resident; 167 (79.5 %) for edematous malnourished and 175 (83.3 %) for severely wasted children. Edematous and severely wasted children were statistically different in age (*P*-Value = 0.0001). The mean age (SD) for edematous malnourished children was 21.7(±12.4) months while for severely wasted, 16.4 (±10.3) (Table 1).

**Table 1:** Socio-demographic characteristics of children with SAM in Southern Ethiopia, 2013/14 (*N* = 420)

Characteristics	Edematous malnutrition (210)	Severely wasted (*n* = 210)	Total (*N* = 420)	*X* ^2^-value	*P*-value
	Number (%)	Number (%)	Number (%)		
Stabilization center					
Karat	126 (60)	130 (61.9)	256 (61)		
Fasha	84 (40)	80 (38.1)	164) (39)	0.09	0.764
Residence					
Rural	167 (79.5)	175 (83.3)	342 (81.4)	0.77	0.388
Urban	43 (20.5)	35 (16.7)	78 (18.6)		
Sex					
Male	114 (54.3)	112 (53.3)	226 (53.8)		
Female	96 (45.7)	98 (46.7)	194 (46.2)	0.01	0.922
Age group (month)					
6–11	43 (20.5)	84 (40.0)	125 (29.8)		
12–23	83 (39.5)	86 (41.0)	171 (40.7)	29.09	0.0001
24–35	48 (22.9)	24 (11.4)	72 (17.1)		
36–47	20 (9.5)	8 (3.8)	28 (6.7)		
48–59	16 (7.6)	8 (3.8)	24 (5.7)		

### Anthropometric information of severely malnourished children

The result showed that baseline mean weight (SD) for edematous malnourished and for severely wasted was statistically different (*P*-Value = 0.0001); 6.89 (±1.82) kg for edematous malnourished children and 5.44 (±1.29) kg for severely wasted children. Similarly, children with edematous malnutrition and severely wasted were statistically different in admission length/height (*P*-Value = 0.0001); with admission mean length/height (SD) of 72.99 (±8.29) cm and 69.15 (±7.06) cm for edematous and severely wasted children respectively. Mean mid upper arm circumferences also statically different in edematous and severely wasted children (*P*-Value = 0.0001; mean MUAC (SD) for Edematous malnourished children was 10.79(±0.51) cm and 10.37 (±0.47) for severely wasted (Table 2).

**Table 2:** Baseline anthropometric measurements of children with SAM Southern Ethiopia, 2013/14 (*N* = 420)

Anthropometric measurements (Mean (SD)	Edematous malnutrition (*n* = 210)	Severely wasted (*n* = 210)	Total (*N* = 420)	T-test value	*P*-value
Weight (kg) Mean (SD)	6.89 (1.82)	5.44 (1.29)	6.16 (1.56)	9.44	0.0001
Length/Height (cm)	72.99 (8.29)	69.15 (7.06)	71.07 (7.68)	5.12	0.0001
MUAC (cm) Mean (SD)	10.79 (0.51)	10.37 (0.47)	10.58 (0.49)	8.67	0.0001

### Treatment outcomes of children with severe acute malnutrition

**Fig. 2:**
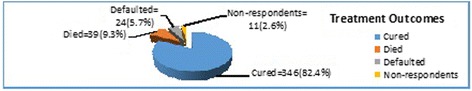
Displayed treatment outcomes of children with SAM managed at Karat and Fasha TFCs. Among 400 total study subjects, 346 (82.4 %) of them recovered from SAM, 39 (9.3 %) died during treatment, 24 (5.7 %) defaulted (left the TFCs before completing treatment) and the rest 11 (2.6 %) were non respondents to treatment or medically transferred to other health facilities

Among the total study subjects, 346 (82.4 %) cured, at Karat TFC, 208 (81.2 %) and at Fasha TFC 138 (84.1 %). Concerning death, 39 (9.3 %) died during treatment, at Karat TFC, 27 (10.5 %) and at Fasha TFC, 12 (7.3.1 %). 24 (5.7 %) defaulted (left the TFCs before completing treatment) and the rest 11 (2.6 %) were non respondents or medically transferred (Fig. 2).

Concerning the primary causes of death, among total 39 deaths, Dehydration secondary to diarrhea (12 (30.8 %)) was the leading cause of death of children with SAM followed by Pneumonia 10 (25.6 %) and the third major cause of death was Malaria 7 (17.9 %). At 5 (12.8 %), Hypoglycemia was the fourth cause of deaths of children with SAM and the rest 5 (12.8 %) deaths were due to Severe Anemia, Septic shock and Hypothermia.

### Survival analysis

The nutritional recovery rate was 3.61 (95 % CI: 3.24–4.0) per 100 person day observations among entire cohorts; 4.18 (95 % CI: 3.59–4.84) and 3.17 (95 % CI: 2.72–3.67) among edematous malnutrition and severely wasted children respectively. The death rate of entire children with SAM was 4.07 (95 % CI: 2.93–5.56) per 1000 person day observations. Actuarial Life Table analysis showed that cumulative probability of nutritional recovery was 99, 91, 77, 56, and 1 % at 5, 10, 15, 20, and 40 days respectively (Table 3).

**Table 3:** Actuarial Life Table analysis of SAM children managed in Karat and Fasha TFCs, Ethiopia, 2013/14

Interval start time	Number entered interval	Number censored	Number exposed risk	Number of recovered	Probability of not recovered	Probability of recovered	Cumulative probability of recovered
0	420	3	418.5	0	0	1	1
5	417	27	403.5	3	0.01	0.99	0.99
10	387	20	377	30	0.08	0.92	0.91
15	337	10	332	52	0.16	0.84	0.77
20	275	1	274.5	75	0.27	0.73	0.56
25	199	3	197.5	101	0.51	0.49	0.27
30	95	4	93	59	0.63	0.37	0.10
35	32	3	30.5	25	0.82	0.18	0.02
40	4	3	2.5	1	0.4	0.6	0.01

### Nutritional recovery time of children with severe acute malnutrition

The median nutritional recovery time of the entire cohort using Kaplan Meier survival analysis was 26 days (95 % CI: 25.1–26.9). Further analysis comparing the median survival time according to stabilization center stratified by malnutrition status showed that there was significant difference in median nutritional recovery time between children with SAM managed at Karat TFC; 27 (95 % CI: 25.1–27.9) and at Fasha TFC; 25 days (95 % CI: 23.8–26.2). But, nutritional recovery time was not significantly different for residence, sex, age (*P*-Value > 0.05) (Table 4).

**Table 4:** Median nutritional recovery time of SAM children by socio-demographic characteristics, Southern Ethiopia, 2013/14

Characteristics	Number (%)	Median recovery time			log rank *X* ^2^-value	*P*-value
		Estimate	95 % CI			
Stabilization center						
Karat	256 (61 %)	27	25.1	27.9		
Fasha	164 (39 %)	25	23.8	26.2	6.77	0.01
Residence						
Urban	78 (18.6 %)	27	25.7	28.3		
Rural	342 (81.4 %)	25	24.1	26.0	0.83	0.361
Sex						
Male	226 (53.8 %)	26	24.7	27.3		
Female	194 (46.2 %)	26	24.9	27.0	0.01	0.982
Age group (month)						
6–23	296 (70.5 %)	26	25.0	27.0		
24–59	124 (29.5 %)	24	22.0	26.0	2.52	0.111

Regarding malnutrition status, there was significant difference in median nutritional recovery time between edematous malnourished 22 days (95 % CI: 20.5–23.5) and severe wasting 29 days (95 % CI: 28.1–29.9). Similarly, there was significant difference in median nutritional recovery time between SAM children who had admission MUAC less than 10.6 cm (27 days, 95 % CI: 25.7–28.2) and SAM patients who had MUAC greater than 10.6 cm (24 days, 95 % CI: 22.8–25.2). There was also significant difference in median nutritional recovery time between SAM children who had admission weight below 6.16 Kg (mean weight) 27 days (95 % CI: 26.1–28.0) and SAM children who had weight above the mean (24 days, 95 % CI: 22.4–25.7). Similarly, there was significant difference in median nutritional recovery time between SAM children who had admission length/height less than the mean (71.1 cm) (27 days, 95 % CI: 26.1–27.9) and SAM patients who had length/height greater than the mean (23 days, 95 % CI: 21.3–24.7) (Table 5).

**Table 5:** Median nutritional recovery time of SAM children by Anthropometric characteristics, Southern, Ethiopia, 2013/14 (*N* = 420)

Characteristics	Number (%)	Median recovery time			log rank *X* ^2^−value	*P*-value
		Estimate	95 % CI			
Malnutrition						
Edematous malnutrition	210 (50 %)	22	20.5	23.5		
Severe wasting	210 (50 %)	29	28.1	29.9	77.23	0.0001
MUAC (cm)						
≤ 10.6	214 (51 %)	27	25.8	28.2		
> 10.6	206 (49 %)	24	22.8	25.2	19.06	0.0001
Weight (kg)						
≤ 6.16	243 (57.9 %)	27	26.1	28.0		
> 6.16	177 (42.1 %)	24	22.4	25.6	9.81	0.002
Length/Height (cm)						
≤ 71.1	258 (61.4 %)	27	26.1	27.9		
> 71.1	162 (38.6 %)	23	21.3	24.7	8.22	0.004

In relation to medical complications, median nutritional recovery time was significantly different for SAM children who had diarrhea (26 days (95 % CI: 25.1–26.9) and their counterparts, 25 days (95 % CI: 23.5–26.5). Likewise, nutritional recovery time was significantly different for children who had dehydration (29 days (95 % CI 25.5–32.3) and their counter parts, 26 days (25.1–26–9). In addition, nutritional recovery time was significantly different between children with anemia (27 days (95 % CI: 24.3–29.7) and without anemia, 26 days (95 % CI: 25.1–26.9). Nutritional recovery time was also significantly different for children who developed complications in TFCs after admission (33 days (95 % CI: 27.3–38.7) and their counterparts, 25 days (95 % CI: 24.1–25.9). However, nutritional recovery time was not significantly associated with complications such as fever, vomiting, malaria, pneumonia, hypothermia, lethargy and NGT-feeding (Table 6).

**Table 6:** Kaplan Meier analysis: nutritional recovery time of SAM children by complications, Ethiopia, 2013/14

Characteristics	Number (%)	Median recovery time			log rank	*P*-value
		Estimate	95 % CI		*X* ^2^-value	
Fever						
Yes	362 (86.2 %)	26	24.8	27.2		
No	58 (13.8 %)	26	24.9	27.0	0.21	0.648
Vomiting						
Yes	330 (78.6 %)	26	23.8	28.2		
No	90 (21.4 %)	26	25.2	26.9	0.94	0.33
Diarrhea						
Yes	223 (53.1 %)	26	25.1	26.9		
No	197 (46.9 %)	25	23.5	26.5	9.76	0.002
Lethargic						
Yes	132 (31.4 %)	26	24.4	27.7		
No	288 (68.6 %)	26	24.9	27.0	0.38	0.539
Malaria						
Yes	121 (28.8 %)	27	25.6	28.4		
No	299 (71.2 %)	26	25.1	26.9	1.32	0.247
Pneumonia						
Yes	86 (20.5 %)	26	24.1	27.9		
No	334 (79.5 %)	26	25.0	26.9	0.03	0.859
Dehydration						
Yes	46 (11 %)	29	25.8	32.3		
No	374 (89 %)	26	25.1	26.9	6.39	0.011
Anemia						
Yes	27 (6.4 %)	27	24.3	29.7		
No	393 (93.6 %)	26	25.1	26.9	5.20	0.023
Inpatient Complication						
Yes	28 (6.7 %)	33	27.3	38.7		
No	392 (93.3 %)	25	24.1	25.9	18.66	0.0001
Naso-gastric feeding						
Yes	61 (14.5 %)	27	25.5	28.5		
No	359 (85.5 %)	26	25.1	26.9	1.72	0.185
Fail to gain appetite at day 4						
Yes	40 (9.5 %)	27	21.5	32.5		
No	380 (90.5 %)	26	25.1	26.9	5.32	0.021
Fail to start losing oedema at day 4						
Yes	14 (6.7 %)	26	25.2	26.8		
No	196 (93.3 %)	23	11.4	34.6	10.46	0.001

**Fig. 3:**
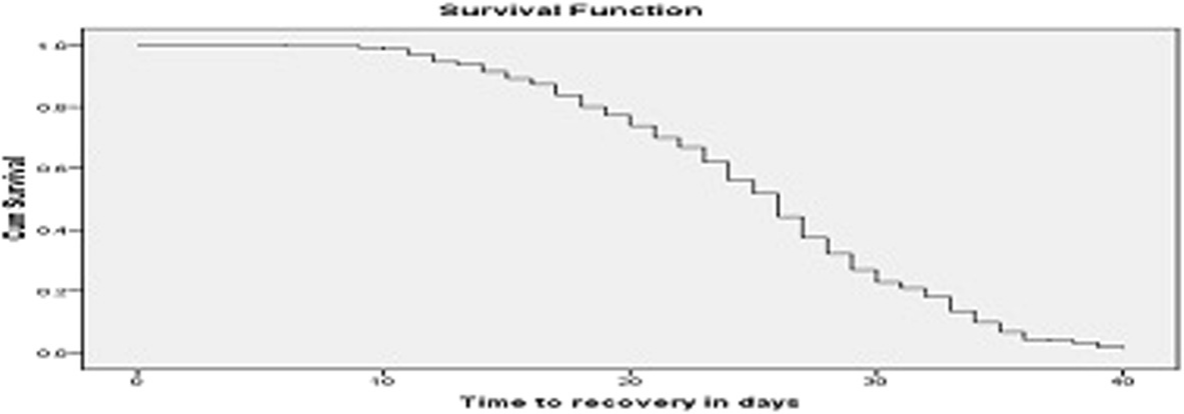
Depicted survival graph for length of stay for entire cohort of children with SAM before getting cured. It illustrated median time of recovery from SAM among children with SAM managed at TFCs. The figure revealed that overall median length of stay for the entire cohorts of children with SAM was 26 days and children with SAM that stayed longer in the TFCs had slimmer chance of getting cured

**Fig. 4:**
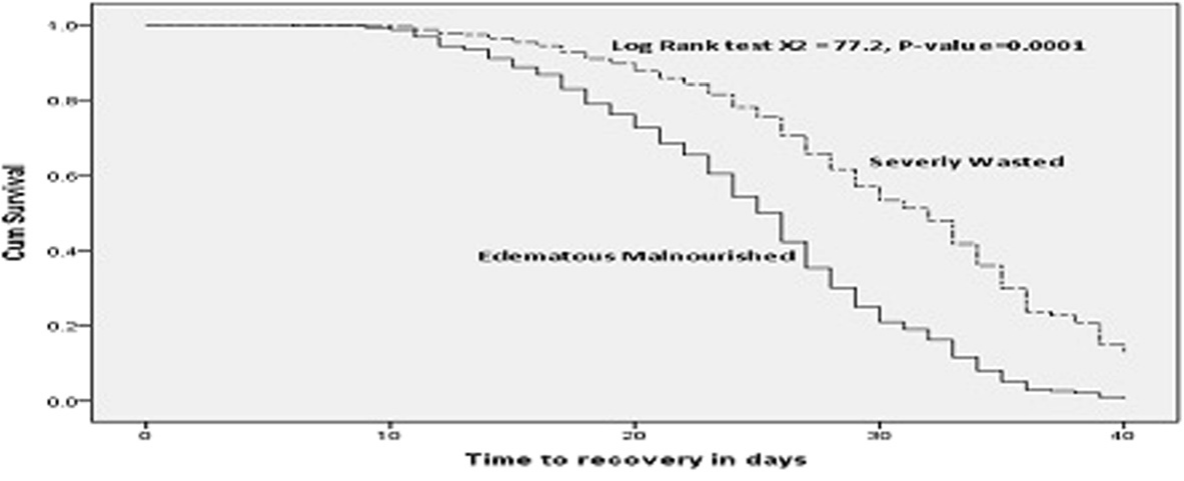
Kaplan meier estimate of survival among children with SAM by malnutrition status, 2013/14. Illustrated Kaplan meier estimate of median recovery time between edematous malnourished and severely wasted children. It showed children who were diagnosed with severely wasted (median recovery time 29 days) stayed longer before recovery than edematous malnourished counter parts (median recovery time 22 days), *P*-Value = 0.000

**Fig. 5:**
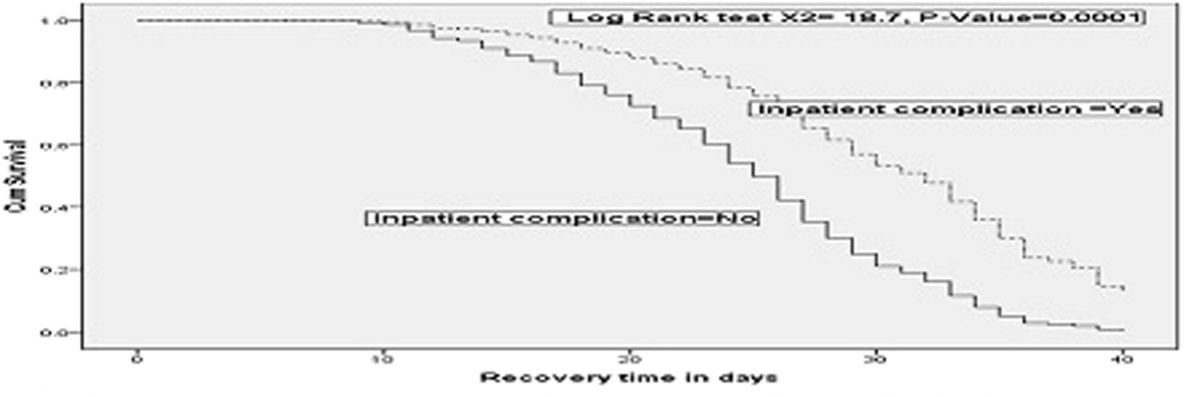
Kaplan meier estimate of survival by complications after admitted to TFCs, Ethopia, 2013/14. Showed Kaplan meier estimate of median recovery time between children developed complications after admitted to TFCs and those children did not acquire complication after admitted to TFCs. The figure portrayed those children who developed complication during management at TFCs, stayed longer (median recovery time 33 days) before recovery than those SAM children who did not develop any complication (median recovery time 25 days), *P*-Value = 0.0001

The overall median length of stay for the entire cohorts of children with SAM was 26 days and children with SAM that stayed longer in the TFCs had slimmer chance of getting cured (Fig. 3). Children who were diagnosed with severely wasted (29 days) on admission stayed longer before recovery than edematous malnutrition (22 days) counter parts, *P*-Value = 0.0001 (Fig. 4). Those children who developed complication at TFCs, stayed longer (33 days) before recovery than those SAM children who did not develop any complication (25 days), *P*-Value = 0.0001 (Fig. 5).

### Predictors of nutritional recovery (survival)

After adjustment, the independent significant predictors of nutritional recovery rate were stabilization center, malnutrition status, weight, MUAC, not loss edema on day four after starting treatment and inpatient complications. Children with SAM who were managed at Fasha TFC were 1.4 times more likely to recover than those who were managed at Karat TFC (AHR = 1.4, 95 % CI: 1.1–1.7) and SAM children who were diagnosed as edematous malnourished were 1.8 times more likely to recover than severely wasted (AHR = 1.8, 95 % CI: 1.3–2.4). Nutritional recovery rate increased by 48.2 % for every one kg (kilo gram) increase in weight (AHR = 1.482, 95 % CI: 1.155–1.902). Similarly, nutritional recovery rate increased by 36.2 % for every one centimeter increase in MUAC (AHR = 1.362, 95 % CI: 1.004–1.847). Children with SAM who did not acquire complications at TFCs were about 2.2 times more likely to recover than those who acquired complications after admission (AHR = 2.2, 95 % CI: 1.4–3.5) and patients who loss edema within four days of treatment were about 2.3 times more likely to recover than those who failed to loss edema within four days of treatment (AHR = 2.3, 95 % CI: 1.1–4.8) (Table 7).

**Table 7:** Predictors of nutritional recovery rate among SAM children managed at Karat and Fasha TFCs, Ethiopia, 2013/14

Covariate	NO. at risk	Cured	Crude HR (95 % CI)	Adjusted HR (95 % CI)
Stabilization center				
Karat	256	208	1	1
Fasha	164	138	1.203 (1.068–1.495)*	1.358 (1.083–1.704)**
Malnutrition				
Edematous	210	175	2.495 (2.002–3.110)***	1.780 (1.333–2.377)***
Severe wasting	210	171	1	1
Weight	420	346	1.157 (1.089–1.231)***	1.482 (1.155–1.902)***
Length/Height	420	346	1.014 (1.001–1.028)*	0.921 (0.876–1.968)
MUAC	420	346	2.094 (1.627–2.694)***	1.362 (1.004–1.847)**
Diarrhea				
Yes	223	174	1	1
No	197	172	1.381 (1.115–1.711)**	1.129 (0.905–1.408)
Dehydration				
Yes	46	28	1	1
No	374	318	1.591 (1.080–2.344)*	1.211 (0.799–1.835)
Anemia				
Yes	27	17	1	1
No	393	329	1.692 (1.037–2.760)*	1.582 (0.962–2.601)
Inpatient Complications				
Yes	28	20	1	1
No	392	326	2.475 (1.567–3.910)***	2.220 (1.392–3.540)***
Not gain appetite day 4				
Yes	40	15	1	1
No	380	331	1.756 (1.045–2.951)*	1.348 (0.772–2.353)
Not start lose oedema on day 4				
Yes	14	8	1	1
No	406	338	2.394 (1.228–4.665)**	2.300 (1.112–4.755)*

NB. * = *P*-Value < .05, ** *= P*-Value ≤ .01 and *** = *P*-Value ≤ .001

## Discussion

This study tried to reveal important information about treatment outcome of children with SAM managed in TFCs, their nutritional recovery time and predictors of nutritional recovery rate. The mean age of children with SAM was 19 months, which was consistent with similar studies conducted in developing countries [10, 16, 17]. Majority of admissions in TFCs were between 6–59 months due to higher risk factors for malnutrition [10, 18, 19].

This study revealed that nutritional recovery rate of SAM children at Karat and Fasha TFCs was above the national minimum standards of cure rate greater than 75 % [10, 20]. The finding is in line with other studies [9, 21, 22]. The cure rate was better than study done in Africa (cure rate of 73 %) [23]. This might be due to difference in settings, caseload and severity of cases. Children with SAM managed at Fasha TFC had a death rate below the national minimum standard of less than 10 % [10]. However, Karat TFC had death rate above the minimum standard for TFC performance which is in line with other studies [9, 16, 17, 22, 23]. The possible reasons might be severe cases were admitted at Karat TFC since it was used as referral health institution. Since most of the deaths occurred within 10 days after starting treatment, it might also be related to institutional factors like “Case-load, staffing, training, quality and availability of supplies” [11, 24]; and this would be the case even in this study, because there were equal numbers three trained health providers in each TFCs even though the patient flow was higher for Karat TFC.

In this study the median nutritional recovery time of the entire cohort were within the accepted national minimum standards of average length of stay in TFC before discharged as cured less than 30 days [10, 20]. This result was in line with other studies conducted in southern Ethiopia and other developing countries [9, 17, 21]. However, a study conducted based on reports of 20 TFCs from 13 African countries revealed varying length of stay from 28–35 days using the new protocol for management of children with SAM at TFCs [23]. This variation might be as a result of difference in settings, severity of cases, caseload, and availability of skilled staffs or due to complications.

The study showed that SAM children who were managed at Fasha TFC had significantly higher nutritional recovery rate than those managed at Karat TFC. This was in concordance with other studies findings where nutritional recovery rate might be different among TFCs [9, 17]. This might be due to late reporting to TFCs by clients; severity of cases since Karat TFC used as referral health institution which was not the case for Fasha TFC. It might be also due to case load since more SAM children were admitted at Karat TFC than Fasha. Studies found out that challenges of TFCs are lack of enough skilled staffs or protocols might not be followed consistently [11, 14, 24].

In this study, age was not independent predictor of nutritional recovery rate. The finding was in line with a study done in Kenya and India [25, 26]. however, it was different from study conducted in southern Ethiopia [9]. This may be due to the fact that they included age less than six months and those above 59 months of age, difference in method of analysis (logistic regression) which does not enable to account for censored cases and time to event. Another study conducted among Malawian children, with SAM also showed that age had slight association with nutritional recovery [27]. This might be due to difference in study design (randomized double blind, controlled trial).

This study indicated that those SAM children who were diagnosed as edematous malnourished were more likely to recover earlier than their severe wasting counterparts. This finding was in line with the findings of other studies, in which those children who were diagnosed with marasmus on admission, stayed longer before recovery than their kwashiorkor counterparts [9, 17, 27]. The nutritional recovery rate of children with SAM was significantly increased as admission weight or MUAC increased. Similarly, studies stated that children with SAM admitted with low MUAC had a longer duration of treatment before recovery than those children admitted with higher MUAC [20, 28, 29]. Correspondingly, a study conducted in Malawi showed that SAM Children that stayed longer in the TFCs had slimmer chance of getting cured [18]. This might be due to early detection, admission with less severe cases and/or types of complications.

This study also revealed that SAM children who did not develop complications at TFCs had significantly higher nutritional recovery rate than their counter parts. The complications might be due to the fact that in both stabilization centers inpatient bed capacity was not still comparable to number of SAM children admitted to the TFCs and even the house structure exposed the patients to complications like hypothermia after admission; some children were managed inside tents which is a great risk to develop complication during treatment. Those children who developed complication after admission return to phase I treatment, which might led to reduction of their nutritional recovery rate [18].

## Conclusions

The findings showed that overall nutritional recovery rate, death rate and length of stay ranged in the accepted minimum international standard set for management of SAM which is cure rate of at least 75 %, death rate less than 10 % and average length of stay of less than 30 days. Children had a lower chance of surviving when managed at Karat than Fasha stabilization center. The probability of surviving gets slimmer with staying longer in therapeutic feeding centers. Therefore, to prevent complications, enhance recovery rate and decrease death rate due emphasis should be given in improving early detection and treatment of severely malnourished children in Ethiopia.

## Abbreviations

AHR: adjusted hazard ratio
EDHS: Ethiopia Demographic and Health Survey
MUAC: mid-upper arm circumference
NGT: naso-gastric tube
SAM: severe acute malnutrition
SC: Stabilization center
TFC: Therapeutic feeding center
WHO: World Health Organization
